# Resistance to retinopathy development in obese, diabetic and hypertensive ZSF1 rats: an exciting model to identify protective genes

**DOI:** 10.1038/s41598-018-29812-w

**Published:** 2018-08-09

**Authors:** Vincenza Caolo, Quentin Roblain, Julie Lecomte, Paolo Carai, Linsey Peters, Ilona Cuijpers, Emma Louise Robinson, Kasper Derks, Jurgen Sergeys, Agnès Noël, Elizabeth A. V. Jones, Lieve Moons, Stephane Heymans

**Affiliations:** 1 Department of Cardiovascular Sciences, Centre for Molecular and Vascular Biology, KU Leuven, Belgium; 2 Department of Cardiology, CARIM School for Cardiovascular Diseases Faculty of Health, Medicine and Life Sciences, Maastricht University, Maastricht, The Netherlands; 3 Laboratory of Tumor and Development Biology, GIGA-Cancer, University of Liège, Liège, Belgium; 4 Department of Genetics and Cell Biology, CARIM School for Cardiovascular Diseases Faculty of Health, Medicine and Life Sciences, Maastricht University, Maastricht, The Netherlands; 5 Laboratory of Neural Circuit Development and Regeneration, Animal Physiology and Neurobiology Section, Department of Biology, KU Leuven, Leuven, Belgium; 6 The Netherlands Heart Institute, Nl-HI Utrecht, The Netherlands

## Abstract

Diabetic retinopathy (DR) is one of the major complications of diabetes, which eventually leads to blindness. Up to date, no animal model has yet shown all the co-morbidities often observed in DR patients. Here, we investigated whether obese 42 weeks old ZSF1 rat, which spontaneously develops diabetes, hypertension and obesity, would be a suitable model to study DR. Although arteriolar tortuosity increased in retinas from obese as compared to lean (hypertensive only) ZSF1 rats, vascular density pericyte coverage, microglia number, vascular morphology and retinal thickness were not affected by diabetes. These results show that, despite high glucose levels, obese ZSF1 rats did not develop DR. Such observations prompted us to investigate whether the expression of genes, possibly able to contain DR development, was affected. Accordingly, mRNA sequencing analysis showed that genes (i.e. *Npy* and crystallins), known to have a protective role, were upregulated in retinas from obese ZSF1 rats. Lack of retina damage, despite obesity, hypertension and diabetes, makes the 42 weeks of age ZSF1 rats a suitable animal model to identify genes with a protective function in DR. Further characterisation of the identified genes and downstream pathways could provide more therapeutic targets for the treat DR.

## Introduction

Globally, prevalence of diabetes has nearly doubled from 4.7% in 1980 to 8.5% of the adult population in 2014^1^. Diabetes can lead to several complications affecting the cardiovascular system, kidneys, nerves, and eyes^2^. Diabetic retinopathy (DR) is one of the most serious complications of diabetes. Prolonged exposure to high blood glucose levels (hyperglycemia) results in severe damage of the retinal vasculature^2–4^. This can lead to blurred vision, dark spots, flashing lights, and eventually total loss of vision. DR is the leading cause of blindness^5^ and makes a big contribution to the total 11.6% of annual health-care costs accounted for diabetes^6^.

Currently, several animal models are being used to study the progression of DR, for example the streptozotocin (STZ)-induced and Akimba mouse models (reviewed in^3^). All these models, however, present serious limitations and they do not reflect all stages of DR progression in humans. In addition, these models do not develop diabetes spontaneously, but are either chemically or genetically induced. For instance, STZ is a toxic substance that is injected in mice or rats to destroy the pancreatic β-cells^3,7^. The Akimba mouse develops DR due to the presence of the human Vascular Endothelial Growth Factor isoform 165 (hVEGF_165_) transgene, which induces an overexpression of VEGF in photoreceptors^8^. Among the few established animal models that develop diabetes spontaneously, the Zucker Diabetic Fatty (ZDF) rat represents an established model for type 2 diabetes. However, ZDF rats did not show any clear sign of DR, i.e. vascular occlusion or regression^9^. Besides the ZDF, the obese ZDF/Spontaneously Hypertensive Heart Failure (SHHF) F1 hybrid (ZSF1) rat, which are the result of a cross between a ZDF female and a SHHF male, develops metabolic complications typical of type-2 diabetes and have a more severe phenotype than the ZDF parental strain. Both obese and lean control ZSF1 rats are hypertensive^10^. However, the obese ZSF1 rats are also affected by diabetic nephropathy (DN), insulin resistance, obesity, hyperinsulinemia, hypercholesterolemia, congestive heart failure, and hypertriglyceridemia^11^. The ZSF1 rat is currently used as a model to study DN, whereas no scientific study on DR performed in these animals has been reported to date. In this study, we extensively investigated whether the ZSF1 rat could represent a suitable animal model to study the pathogenesis of DR. In order to assess the retinal vascular changes caused by diabetes, such as arteriolar tortuosity, obese and lean control ZSF1 rats were subjected to Heidelberg Retina Angiography (HRA) and additional histological analysis over a period of 42 weeks. We further examined the potential combined effect of chronic diabetes, obesity and hypertension on vasculature of retinas isolated from 6 and 42 weeks old obese and lean control ZSF1 rats, by assessing vascular density, pericyte coverage and number of microglia on whole mounted retina. The thickness of the neural retinal layers was assessed by Optical Coherence Tomography (OCT) over a period of 35 weeks. However, no differences were detected between obese and lean ZSF1 rats.

Despite the increase in vascular tortuosity, obese ZSF1 rats did not develop DR. The absence of a DR phenotype would suggest the existence of a protective gene expression profile in these rats. Consistently, deep sequencing analysis of mRNA isolated from retinas of 6 and 42 weeks old obese and lean ZSF1 rats, revealed the upregulation of several genes with a potential protective function, i.e. Neuropeptide Y (*Npy*) and several crystallin genes, at 42 weeks. Whereas genes, previously described to drive vascular inflammation, such as Intercellular Adhesion Molecule 1 (*Icam1*) and Toll-like receptor 4 (*Tlr4*) were downregulated in retinas from obese rats 42 weeks of age.

A deeper understanding on the role of *Npy* and the crystallin genes in the retina following stressful conditions such as diabetes could be beneficial for developing better tools to improve the condition of patients affected by DR and other diabetes related ocular complications.

## Results

### Arteriolar tortuosity is increased in obese ZSF1 rats over time

**Figure 1:**
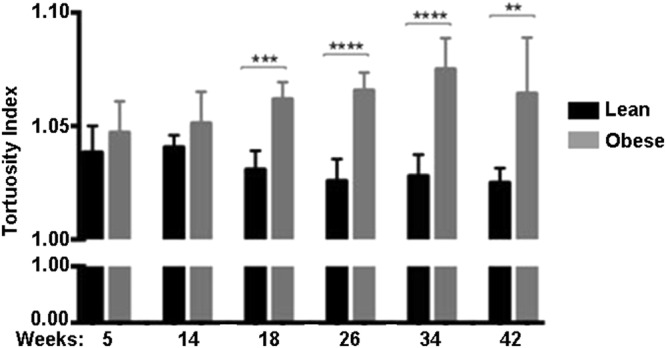
Arterial tortuosity index of the retinal vasculature from lean and obese ZSF1 rats, between 5 and 42 weeks old. Tortuosity index of the retinal arteries was increased in obese as compared to lean ZSF1 rat retinas of 18, 26, 34 and 42 weeks of age. No difference in arteriolar tortuosity was detected in rats of 5 and 14 weeks of age. All values are mean ± SD, **P < 0.01; ***P < 0.001; ****P < 0.0001.

In the clinic, arteriolar tortuosity is routinely used as a marker to predict pathological neovascularization and rapid DR progression^12^. Therefore we assessed arteriolar tortuosity index in lean and obese ZSF1 rats over a period of 6 to 42 weeks of age. As shown in Fig. 1, the tortuosity index significantly increased in obese rats compared to their lean controls at 18, 26, 34 and 42 weeks, but did not differ when comparing lean and obese ZSF1 rats at 5 and 14 weeks of age. Increase in weight and blood glucose levels was confirmed in obese ZSF1 rats at 18, 22 and 42 weeks as reported in Supplementary Figure 2A,B.

### Retinal vascular density and pericyte coverage are not affected by diabetes or age

**Figure 2:**
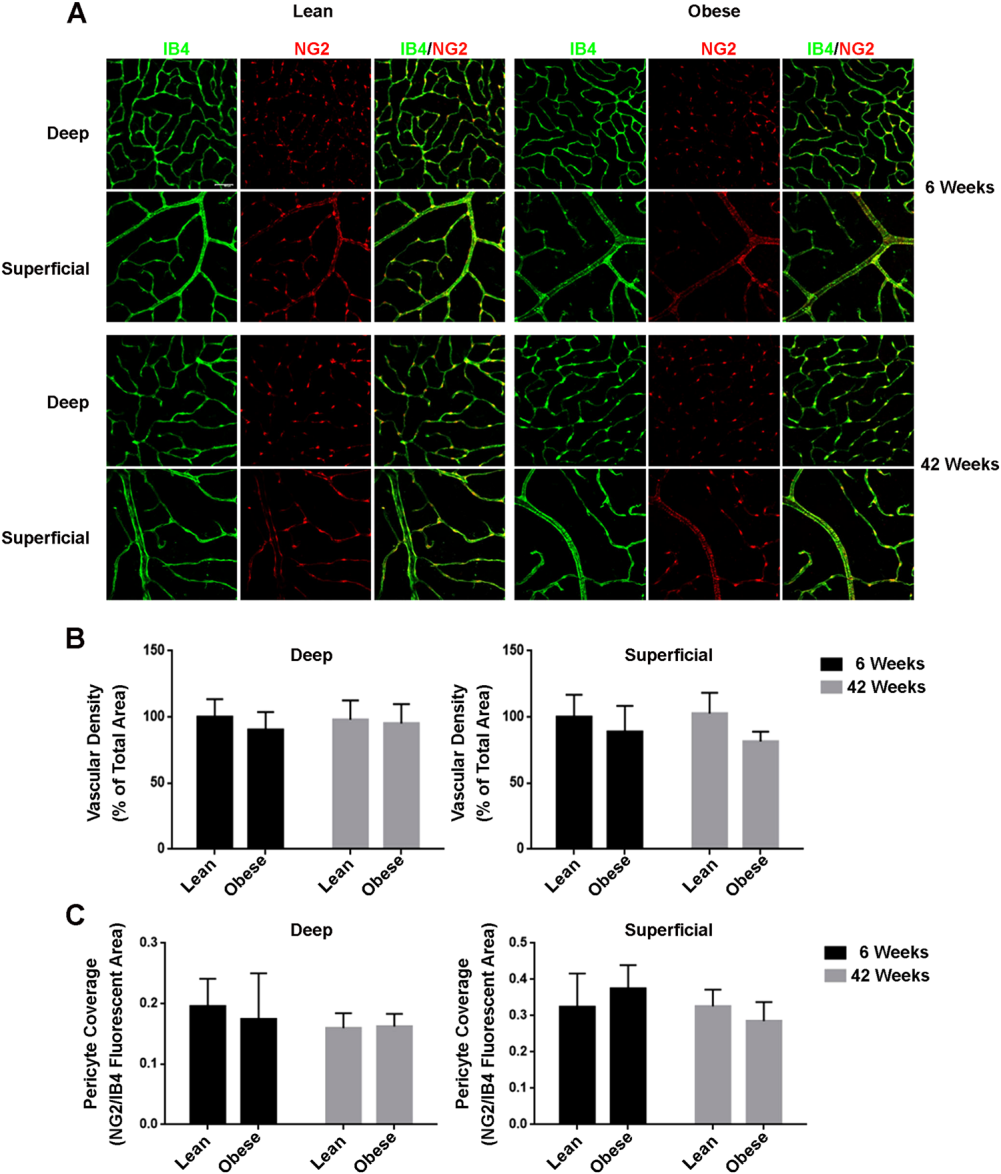
Vascular density and pericyte coverage of retinal vasculature from 6 and 42 weeks old lean and obese ZSF1 rats. (**A**) IB4 (green), NG2 (red) and a merge signal of both the deep and superficial retinal vascular plexus. Scale = 50 µm. (**B**) Quantification of vascular density of the deep and superficial plexus shown in percentage of IB4 signal of the total area (n = 6–7 per data point). (**C**) Quantification of pericyte coverage of the deep and superficial plexus shown as ratio of the NG2 and IB4 positive areas (n = 5–7 per data point). All values are mean ± SD.

Besides vessel tortuosity, two characteristics of DR are pericyte dropout and pathological neovascularization. Specifically, pericyte dropout leads to capillary occlusions followed by hypoxia^13,14^. In the initial phase, blood vessels retract due to vessel instability caused by the loss of pericytes^15^. This leads to hypoxic areas, which in turn increases the expression of HIF-1 and subsequent VEGFA, resulting in pathological neovascularisation^16,17^. Remarkably, deep and superficial vascular density and pericyte coverage did not differ between lean and obese animals at 6 and 42 weeks of age, but also there was no time-induced difference in obese and lean control ZSF1 rats at 42 weeks compared to those at 6 weeks (Fig. 2B,C). These results suggest that the vascular density and pericyte coverage are not affected by both, age and diabetes in ZSF1 rats.

### The number of microglia is affected by age, but not by diabetes

**Figure 3:**
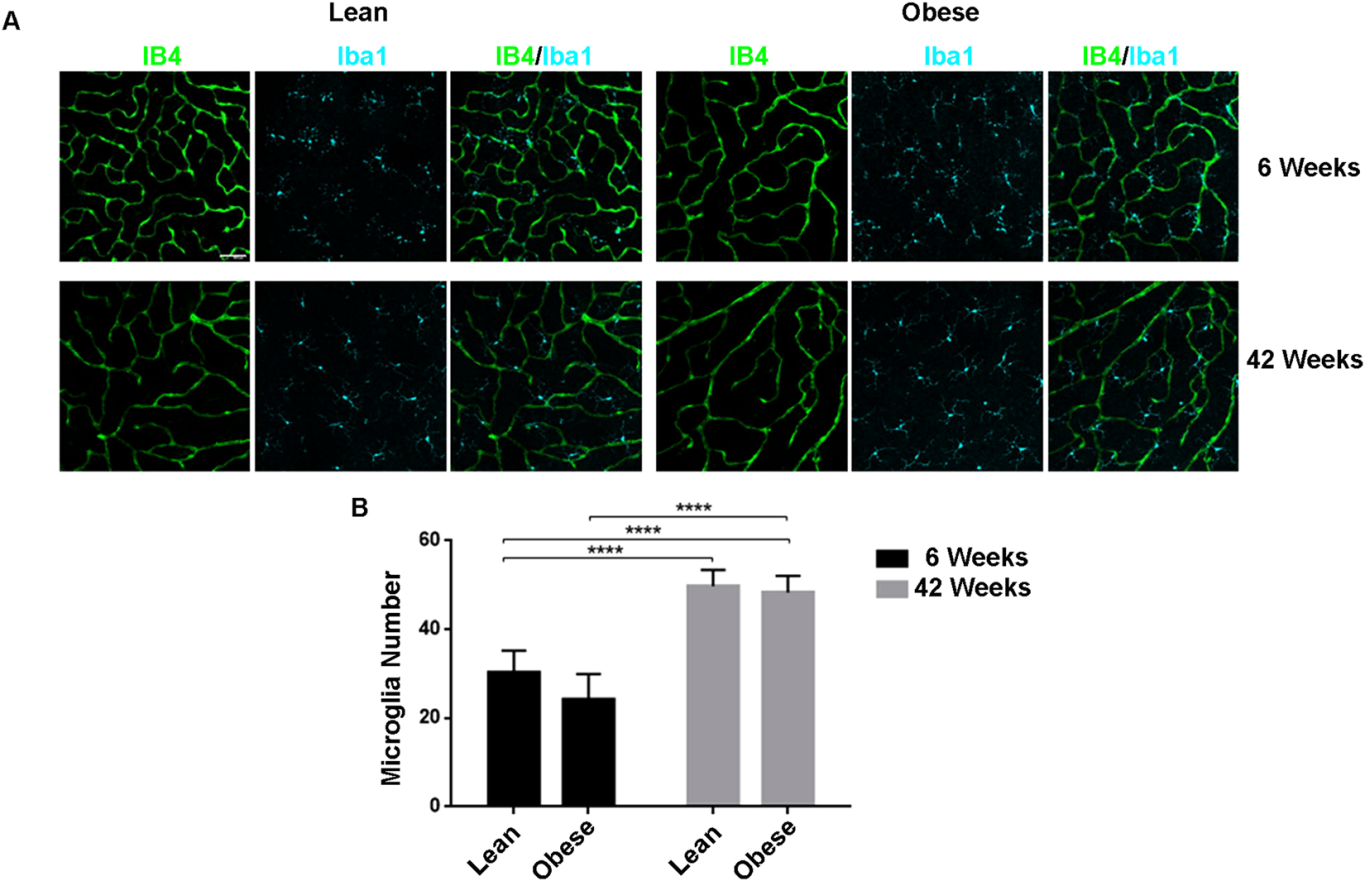
Number of microglia located in the retinas from 6 and 42 weeks old lean and obese ZSF1 rats. (**A**) IB4 (green) and Iba1 (cyan) and merge signal of the deep retinal vascular plexus. Scale = 50 µm. (**B**) Quantification of microglia number counted in the intermediate and deep vascular plexus (n = 5–7 per data point). All values are mean ± SD, ****P < 0.0001.

The activation of several signalling pathways during DR results in increased inflammation^4^. Microglia constitute the resident immune cells of the central nervous system^18^ and they seem to play an important role during DR progression^19–21^. The number of Iba1-stained microglia was significantly higher in the retinas of both lean and obese ZSF1 rats at 42 weeks as compared to lean and obese ZSF1 rat at 6 weeks (Fig. 3A,B), which is in line with an age-dependent increase^21^. However, the number of microglia did not differ between lean and obese ZSF1 either at 6 and 42 weeks of age (Fig. 3B), indicating that diabetes did not impact the microglia cell number in obese ZSF1 rat retinas.

### The morphology of the vasculature is affected by age, but not by diabetes

**Figure 4:**
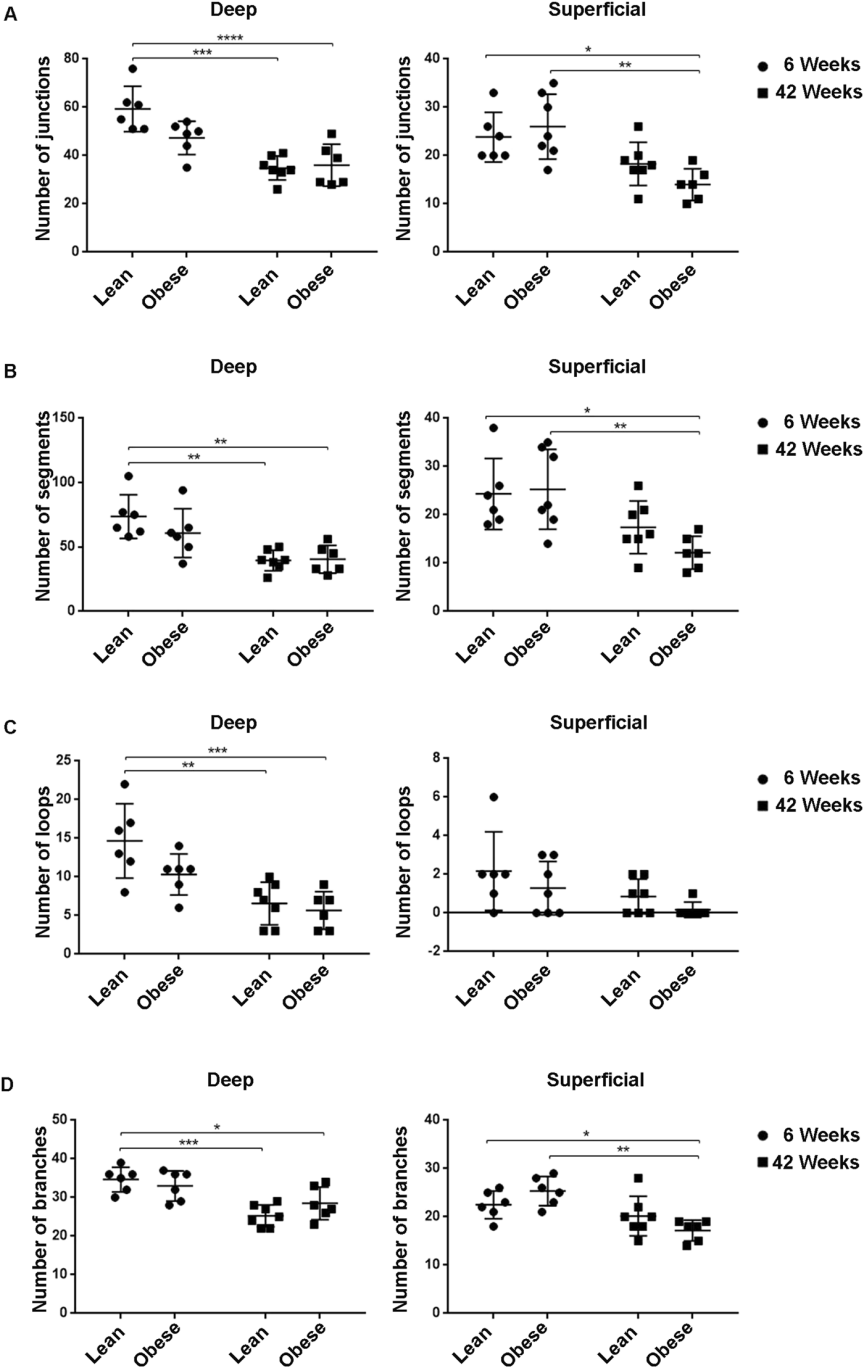
Quantification of vascular network morphology of the vascular deep and superficial plexus of retinas from 6 and 42 weeks old lean and obese ZSF1 rat. Number of junctions (**A**), segments (**B**), loops (**C**) and branches (**D**) in the deep and superficial retinal vascular plexus (n = 5–7 per data point). All values are mean ± SD, *P < 0.05; **P < 0.01; ***P < 0.001; ****P < 0.0001.

Next, we determined whether obese ZSF1 rats showed any changes in the morphology of their retinal vascular network, stained with IB4, as compared to their lean controls. An illustration of junctions, segments, loops and branches is reported in Supplementary Figure 2. In the deep retinal vascular plexus, the number of junctions, segments, loops and branches were significantly decreased in 42 weeks old lean and obese rats as compared to the 6 weeks old lean and obese ZSF1 rats, respectively (Fig. 4A–D; Deep). Also, in the superficial vascular plexus, the number of junctions, segments and branches were significantly decreased in the 42 weeks old obese ZSF1 rats compared 6 weeks old obese ZSF1 rats (Fig. 4A,B,D; Superficial). No significant difference was found in the number of loops in the superficial plexus between the groups (Fig. 4C; Superficial). Finally, no significant difference was found in the number of junctions, segments, branches and loops in the superficial and deep plexus of retinal vasculature between lean and obese ZSF1 rats at 6 and 42 weeks respectively. Overall, these results indicate that the vascular morphology in the retina is affected by age, but not by diabetes in both deep and superficial plexus. Functional analysis, i.e. OCT scan, of the retina was also performed. As shown in Supplementary Figure 3, no differences in neural retina were found between obese and lean rats.

### Upregulation of Npy and several crystallin genes in retinas from obese ZSF1 rats of 42 weeks of age

**Figure 5:**
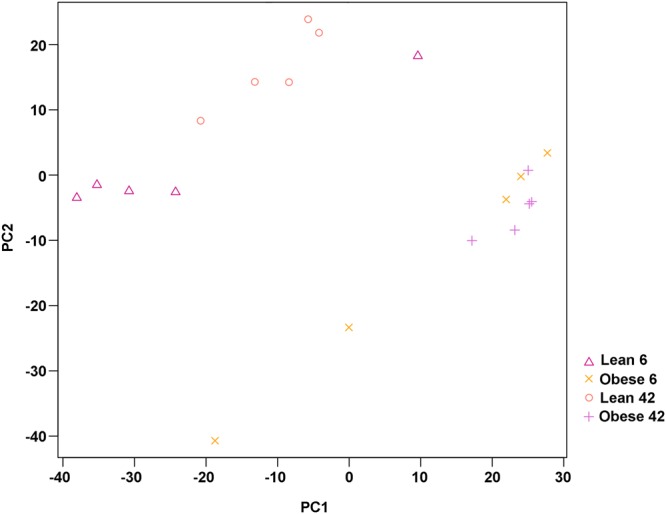
Principal component analysis (PCA) plot based on the first two (PC1–PC2) components of mRNA from retinas from 6 and 42 weeks old lean and obese ZSF rats.

To identify genes able to prevent the onset of DR in obese ZSF1 rat, we performed sequencing of mRNA of retinas from 6 and 42 weeks old lean and obese, ZSF1 rats. Principal Component Analysis (PCA) resulted in a clear separation of lean from obese ZSF1 rats (Fig. 5). Although less pronounced, a separation based on age could be also distinguished within the lean and obese groups, i.e. 6 versus 42 weeks old lean and 6 versus 42 weeks old obese ZSF1 rats (Fig. 5).

**Figure 6:**
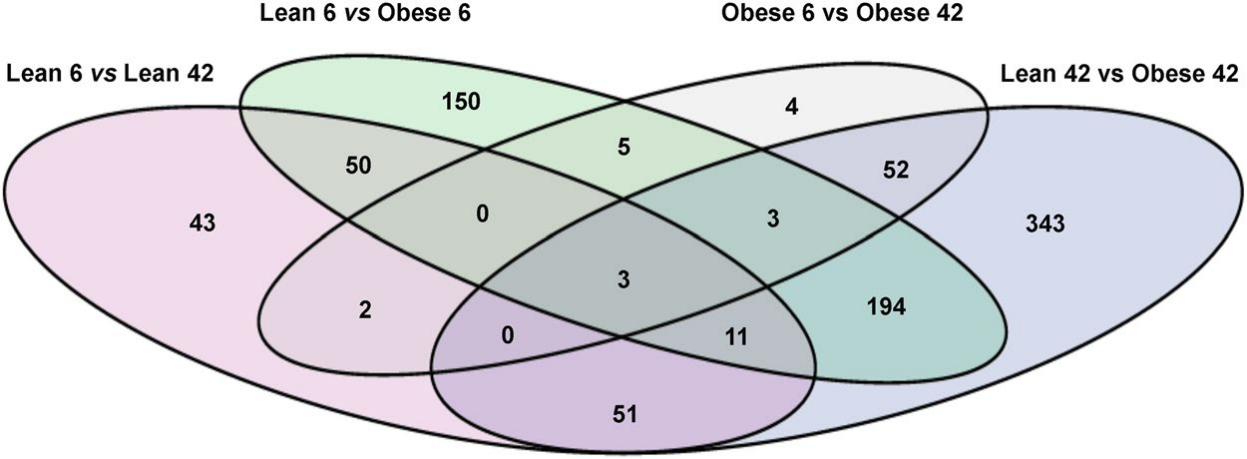
Venn diagram showing overlap between the differentially expressed genes (DEG) found in different comparisons, i.e. by age (Lean 6 vs Lean 42, purple and Obese 6 vs Obese 42, gray) and diabetes and metabolic syndrome (Lean 6 vs Obese 6, green and Lean 42 vs Lean 42, blue). The cut-offs for the DEGs were set to a Fold Change < or > 1,5 and (FDR) < 0.05. The numbers in the circle depict the number of DEGs found for that specific condition: i.e. in the bottom circle 51 DEGs are found in both Lean 6 vs Lean 42 and Lean 42 vs Obese 42.

We performed statistical analysis on all genes expressed between the different groups. A gene was considered to be expressed when at least 5 reads were found aligned in at least all samples of one of the groups. In Fig. 6, we report the differential expressed genes (DEGs) found for all the comparisons. Changes in the expression of a total of 911 protein coding genes were identified (FC >1.5 False Discovery Rate (FDR) < 0.05). A large majority of the DEGs identified per comparison was unique to that specific comparison, i.e. the DEGs were not significantly regulated in the other comparisons. Ingenuity Pathway Analysis (IPA) analysis showed the differently regulated pathways within the 4 comparisons: lean vs obese 6 weeks old, lean vs obese 42 weeks old, lean 6 vs lean 42 weeks old, obese 6 vs obese 42 weeks old (Supplementary table 1–4). Noteworthy, the leptin signalling pathway appeared to be differently regulated in the obese 6 and obese 42 weeks old (Supplementary table 4), confirming the metabolic phenotype affecting old obese ZSF1 rats (Supplements, table 4).

**Figure 7:**
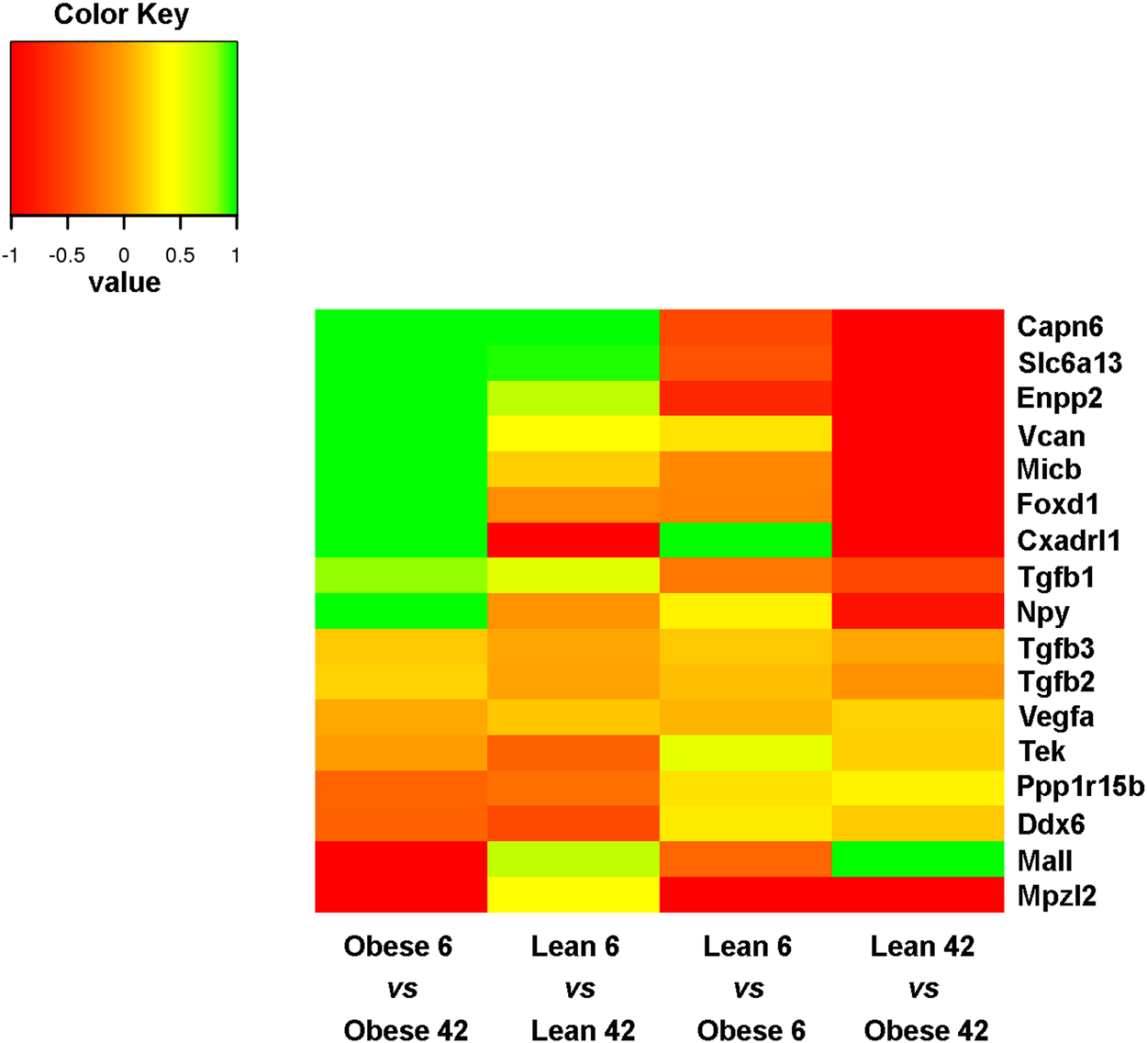
Heat map displaying fold changes of the differential expressed genes. The colors are ranging from red (−1 <= log2 Fold Change (FC)) to orange (FC ~ 0) to green (FC >= 1).

**Figure 8:**
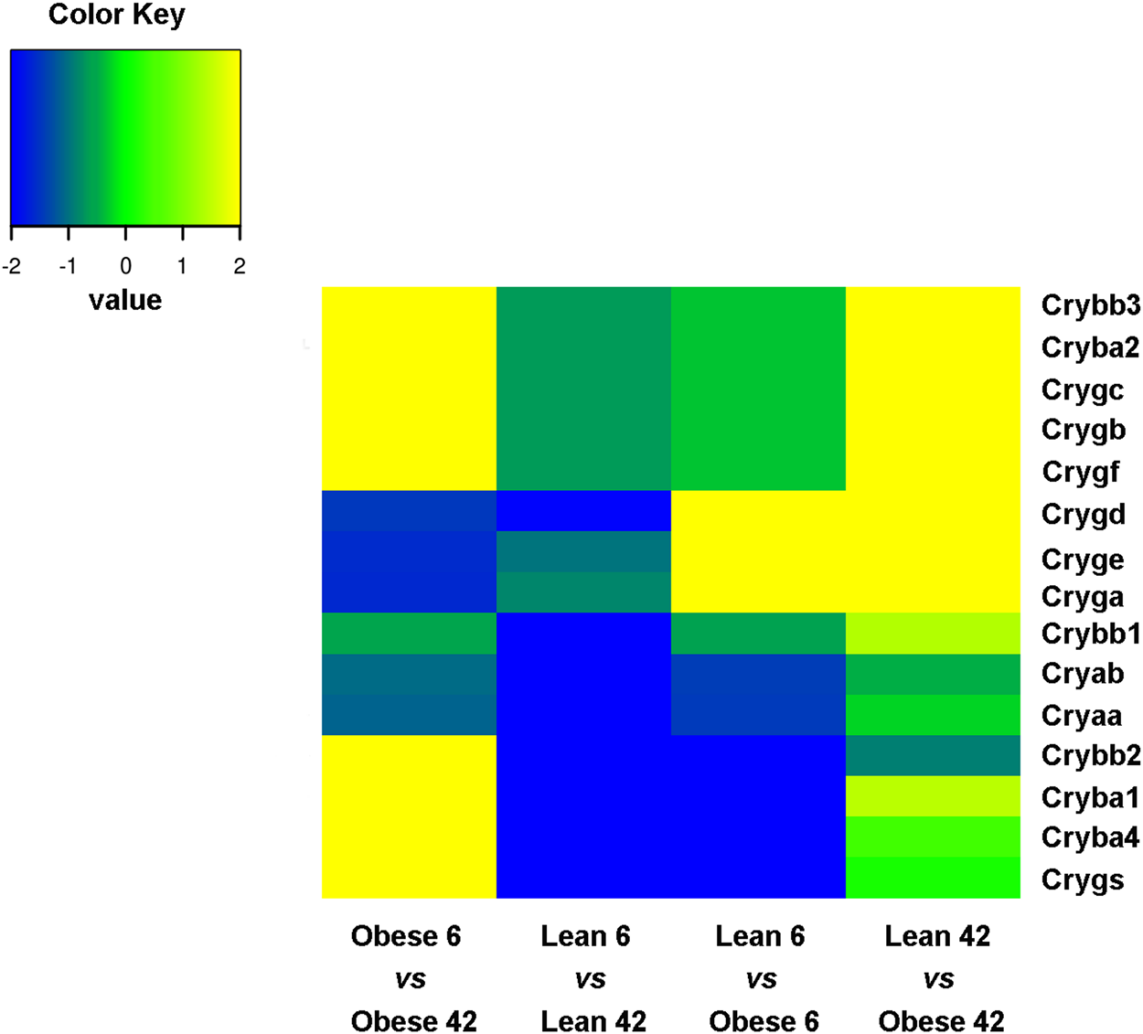
Heat map displaying fold changes of the differential expressed crystallin (Crys) genes. The colors are ranging from blue (−2 <= log2 Fold Change (FC)) to green (FC ~ 0) to yellow (FC >= 2).

**Figure 9:**
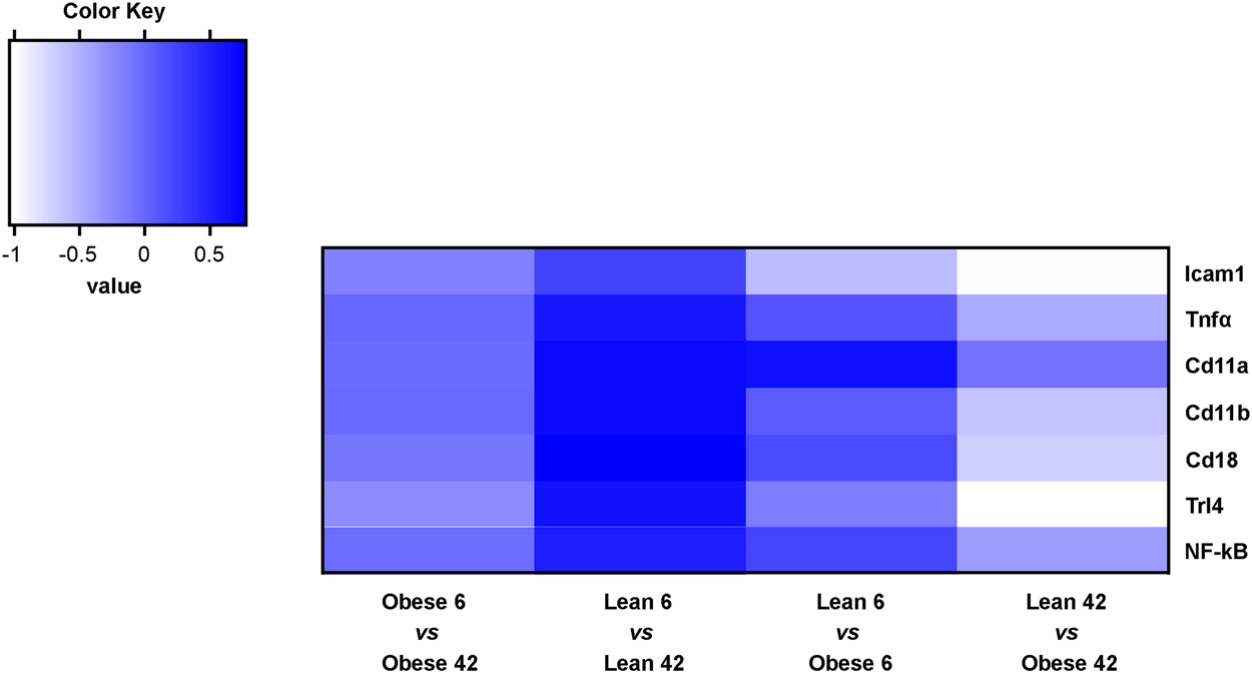
Heat map displaying fold changes for genes indicated. The colors are ranging from white (−1 <= log2 Fold Change (FC)) to light blue (FC = ~ 0) to dark blue/violet (FC >= 1).

Among genes only upregulated in the retinas from 42 weeks old ZSF1, we found Versican (*Vcan*), Forkhead Box D1 (*Foxd1*), MHC Class I Polypeptide-Related Sequence B (*Micb*) and *Npy*, whereas *Cxadrl1*, as Immunoglobulin Superfamily Member 11 (*IGSF11*), and Mal-like (*Mall)* were downregulated (Fig. 7). Remarkably, among the top regulated genes in retina from 6 vs 42 weeks old obese ZSF1, were several members of the crystallin gene family (Fig. 8). Among them, *Crybb3*, *Cryba2*, *Crygc*, *Crygb*, *Crygf*, *Crybb2*, *Cryba1*, *Cryba4*, *Crygs*, were upregulated in obese 42 as compared to obese 6 weeks old. However, *Crybb3*, *Cryba2*, *Crygc*, *Crygb*, *Crygf*, *Cryba1* were only upregulated in obese 42, indicating that their expression was likely affected by diabetes and metabolic diseases. On the other hand, *Icam1* and *Trl4*, both known to play a determinant role during vascular inflammation, were both downregulated in retinas from obese rat 42 weeks old (Fig. 9).

## Discussion

The ZSF1 rat, despite being obese, hypertensive, diabetic and hyperlipidemic, did not present any clear vascular defects in their retinas that could be attributed to progressive DR. Although vascular tortuosity increased in the diabetic rats over time, vascular density and pericyte coverage did not differ between diabetic and non-diabetic rats of 6 and 42 weeks of age. Microglia number and vascular network morphology differed in aged rats, but were unaffected by diabetes. Thickness of neural retina layers were also assessed by OCT scan, however no differences were found at the different analysed time points, in lean and obese rats. Absence of damage in the retinas from diabetic aged ZSF1 rats offered us the existing opportunity to investigate on possible molecular mechanisms that would prevent these animals from developing DR. We, therefore, performed mRNA sequencing in order to identify genes differentially expressed in retina isolated from lean and obese ZSF1 rats 6 and 42 weeks of age with a possible protective role against DR.

The RNA sequencing revealed that 85 genes were upregulated in the retinas from 42 weeks old when compared to their lean control ZSF1 rats. Among the regulated genes, we found genes known to play a role in angiogenesis, inflammation, cellular stress and death. We found that genes involved in the process of angiogenesis, i.e. VCAN and Foxd1, were upregulated. In addition, the expression of the Micb gene, whose increase has been associated with degeneration of retinal ganglionic cells (RGC) and development of glaucoma^22^, was upregulated in retinas from 42 weeks old ZSF1 rats. On the other hand, Cxadrl1, important for excitatory synaptic localization and plasticity, and Mall, encoding a component of raft mediated trafficking in endothelial cells, were both downregulated. Although changes in expression of the above mentioned genes, together with increased vascular tortuosity, would suggest that an initial vascular and neuronal damage of the retinas in ZSF1 rats 42 weeks of age is occurring, we also found that several genes with a potential protective function, i.e. *Npy* and several crystallin genes, were significantly upregulated in retinas from 42 weeks old ZSF1 rats. Conversely, genes involved in vascular inflammation, like *Icam1* and *Trl4*^23,24^, were downregulated in in retinas from 42 weeks old ZSF1 rats.

Npy was first isolated in 1982 and is highly conserved between species^25^. In the retina, Npy seems to be required during development being expressed in different cell types^26^. Specifically, the neuroprotective role of Npy against excitotoxicity has been largely documented^27–29^. As such, Npy was shown to reduce the [Ca^2+^]_i_ increase in rat neurons preventing their death^30^. The activation of several Npy receptors has been reported to protect retinal cells from necrosis or glutamate induced cell death^31^. *Npy* and its receptors are expressed in retinal endothelial cells, likely playing a role in retinal related vascular diseases^30^. In support of this, few studies have shown that Leucine to Proline polymorphism in the Npy gene was related to an increased predisposition to develop diabetic retinopathy in type 2 diabetic patients^32–34^. However, results on the role of Npy in oxygen-induced retinopathy model have been controversial. Whereas two studies^33,35^ suggested a positive role for Npy in the progression of neovascularization during DR, Schmid and collaborators^36^ reported a decreased expression level of Npy in a similar model. Therefore, further research is still needed to clarify the exact contribution of Npy in retinal vascular diseases.

Several crystalline genes were also found to be upregulated in retinas of obese ZSF1 when compared to lean 42 weeks of age. The crystallin genes were first described to encode structural proteins of the lens^37^, but they are also expressed in other tissues and organs such as the retina^38^, heart^39^ and skeletal muscle^40^. The crystallins are divided in two families: the α-crystallin belonging to the small heat shock proteins (sHSP) and the β- and γ-crystallins forming the β/γ superfamily. Besides their function in lens transparency and reflex index, the α-crystallins are induced in response to stress and injury, displaying molecular chaperone and anti-apoptotic activity^41^. The β/γ crystallins regulate vascular remodelling during eye development and RCG axon regeneration^42^. Crystallin gene expression is altered upon DR^38^, but their exact function during this process remains controversial. Although few studies have suggested a protective role of αB-crystallin genes against pathological neovascularization of the retina during diabetes, one study reported that delivery by intravitreous injection of recombinant adenovirus expressing αA-crystallin prevented vascular leakage and decreased pericyte loss in STZ-induced mouse model of diabetes^43–46^. The majority of those studies, however, investigated the role of α-crystallin genes during DR, whereas little is still known on the role of β/γ crystallins. From our RNA sequencing analysis, it emerges that diabetes and the metabolic syndrome induce the expression of crystallin β (i.e. Crybb3, Cryba2, Cryba1) and γ (i.e. Crygc, Crygb, Crygf) in the retina, whereas we did not observe any significant effect on α crystallins.

Noteworthy, β and γ crystallin genes are upregulated in the retina of Nuc1 mutant rats as compared to wild type littermates^47^. Nuc1 mutants are characterized by a spontaneous mutation that affects neuronal and vascular remodelling and retinal function^48^. In particular, astrocytes at the vascular front seem to express β and γ crystallins together with VEGF. Moreover, in human persistent fetal vasculature (PFV) disease, in which the hyaloid vasculature does not regress normally, astrocytes expressed β and γ crystallins^47^, suggesting a role for those crystallins in vascular stabilization in the eye. Besides being vasculoprotective, β/γ crystallins may also modulate axon regeneration^49^. Β/γ crystallins induce ciliary neurotrophic factor (CNTF) and brain derived neurotrophic factor (BDNF) both *in vitro* and *in vivo*^49^. In particular Crybb2 is secreted by cultured retinas during axon regeneration, and induces axon elongation in cultured RCG axons^42^. In conclusion, β/γ crystallin genes, which are upregulated in the retinas of 42 weeks old obese ZSF1 rats, might play a role in vascular stabilization and neuronal survival, preventing ZSF1 rats from developing DR.

Vascular inflammation and leukostasis are early events in diabetic retinopathy with serious functional consequences. During DR leukocytes adhere to endothelial cells, causing vascular occlusion, macrophage accumulation and vascular tissue damage^50^. Several are the genes involved in these processes. Among them, Tumor Necrosis Factor α (Tnfα) modulates endothelial cell permeability and adhesion molecule expression^51^. Also, the expression of CD11a, CD11b, and CD18 integrins was shown to be increased on the surface of neutrophils from diabetic rats^50^. Proteins such as, Tnfα, NF-kB are Trl4 have been extensively shown to regulate the inflammatory response^23^. Finally Icam1 inhibition was reported to prevent diabetic retinal leukostastis and blood-retinal barrier breakdown^24^. Therefore, we assessed the expression of all these vascular inflammation markers and to our surprise we found that *Icam1* and *Trl4* were downregulated in in retinas from 42 weeks old ZSF1 rats, likely contributing to the protective mechanisms by reducing leukostasis and inflammation.

Although the diabetic, hypertensive, obese and hyperlipidemic ZSF1 rat does not develop overt DR, it represent a useful model to identify new molecular signalling pathways beneficial to prevent the onset of DR. Remarkably, RNA sequencing analysis led us to identify *Npy*, β/γ crystalline, *Icam1* and *Trl4* genes that are differently expressed in lean and obese ZSF1 rats and could play a role in protecting old obese ZSF1 rats from developing DR. The roles that *Npy* and β/γ crystallins play in vascular remodelling and axon regeneration, and that *Icam1* and *Trl4* play in vascular inflammation, make them interesting candidates for new studies to target a complex ocular disease such as DR. Further characterization of the molecular mechanisms regulated by β/γ crystallins and *Npy*, *Icam1* and *Trl4*, whose activation might be protective against DR, could possibly help us to design novel and more effective therapeutic strategies to improve the clinical outcome of patients affected by DR.

## Methods

### Animals

All animal procedures conformed to the relevant guidelines and regulations of, and were approved by, the Animal Welfare Committee of the KU Leuven University. Male obese ZSF1 rats (ZSF1-Lepr^fa^Lepr^cp^/Crl) (Charles River Inc.) were used in this study. Male lean, non-diabetic, non-obese, but hypertensive ZSF1 rats were used as control. Rats were sacrificed at 6 and 42 weeks of age. The weight of each overnight fasting rat was recorded. Diabetes was confirmed after blood collection from rat tails at different time points (18, 22 and 42 weeks) and glucose levels were determined with an automated glucose analyzer device (Glucometer, Menarini Diagnostics).

### Heidelberg Retinal Angiography

Rats were anesthetized by intraperitoneal injection of ketamine (Ketalar, 10 mg/mL) at a dose of 6.0 mg/kg and medetomidine hydrochloride (Domitor, 1.0 mg/mL) at a dose of 0.4 mg/kg. Rats were injected intraperitoneally with 1 mL fluorescein sodium salt (Sigma, F6377-100G) 10% solution in saline. After the experiment, rats were awaken by intraperitoneal injection of atipamezole hydrochloride (Antisedan, 5 mg/ml) at a dose of 0.5 mg/kg. Images were taken using a Heidelberg Retina Angiograph 2 (HRA2) (Heidelberg Engineering GmbH) according to the manufacturer’s recommendations. Tortuosity index was calculated from these photographs using the ImageJ software as previously described^52^.

### Retina Isolation and Immunofluorescence

Retina isolation and staining were carried out as previously described^53^. Blood vessels were stained with 20 µg/mL Isolectin GS-IB4 (IB4) (I21411, Thermo Fisher). Pericytes were stained with 5 µg/mL primary antibody Mouse anti-NG2 (Cat. No. NG2 37-2700 Invitrogen) and microglia were stained with 2 µg/mL primary polyclonal antibody Rabbit anti-Iba1 (019_19741, Wako Laboratory Chemicals). 1:100 Goat anti-Mouse Alexa 568 (A11031, Molecular Probes) and 1:100 Donkey anti-Rabbit Alexa 647 (A31573, Invitrogen) were used as secondary antibodies. Photos were taken using a Leica DFC350 FX digital camera or Leica TCS SPE confocal.

The images were processed and analyzed in ImageJ. The superficial and deep vascular plexus were separated based on the IB4 images. All analyses were performed on 20X magnification images with ImageJ Angiogenesis Analyzer. This tool allows detailed quantification of several vasculature structures, such as number of junctions, segments, loops and branches as shown in Supplementary Figure 2. Junctions were defined as meeting points of segments and/or branches. Segments were described as elements between two junctions and branches are elements between a junction and extremity. Loops were defined as areas enclosed by segments^54^. The percentage of IB4 positive areas was evaluated to determine vascular density. The percentage of NG2 positive areas was measured and the ratio of IB4 and NG2 positive areas was calculated to determine pericyte coverage. For area calculations, the images were quantified by ImageJ Area Fraction analysis. Microglia were quantified by ImageJ Cell Counter analysis.

### Optical Coherence Tomography

To assess thickness of the retinal layers and retinal morphology, a spectral domain optical coherence tomography (SD-OCT) system (Envisu R2210, Bioptigen, Morrisville, NC, USA) was used. To evaluate retinal morphology and neural retinal thickness an InVivoVue Diver 2.2 software (Bioptigen) was used as previously described in^55^.

### RNA isolation and integrity

RNA was isolated from the ZSF1 retinas using the mirVana™ miRNA Isolation Kit (Cat. No 1560, Ambion) according to the mirVana™ miRNA Isolation kit protocol. In a 20 µL reaction volume, 1 µg RNA per sample was reverse transcribed into cDNA via the miScript II RT Kit (Cat. No. 218160, Qiagen), according to the manufacturer’s instructions using the 5x miScript HiFlex Buffer.

The integrity of the RNA from each sample was scored on the Agilent 2100 Bioanalyzer (Agilent) by using an Expert Eukaryote Total RNA Pico chip according manufacturer’s protocol. Samples that had an RNA Integrity Number (RIN) value of over 6 were subsequently used for mRNA sequence library generation.

### mRNA sequencing library generation

The mRNA sequencing library was generated using TruSeq mRNA sample preparation kit (Illumina) according to manufacturer’s protocol. In short, mRNA was enriched using magnetic beads coated with poly-dT, followed by fragmentation. The fragmented mRNA- enriched samples were subjected to cDNA synthesis by reverse transcriptase, followed by dA-tailing and ligation of specific double-stranded bar-coded adapters. Subsequently, a 15 cycles library amplification was performed and after clean-up, the sizes of the libraries were determined on an Agilent 2100 Bioanalyzer (Agilent) via an DNA 1000 chip according manufacturer’s protocol. Pooled libraries consisting of equal molar samples were sequenced on a high-output 75 bp single read on the NextSeq500 (Illumina).

### Total RNA analysis pipeline

The analyses of sequencing datasets were performed as earlier described^56^. In short, reads were aligned to the rat rn6 reference genome using TopHat^43^ and exonic reads were summed per transcript and transcripts were referred to as being expressed when at least five aligned reads were present in all samples of at least one of the groups.

### Pathway analysis

The differentially transcribed genes for the obese, i.e. 6 and 42 weeks, or lean, i.e. 6 and 42 weeks, ZSF1 rats were investigated for over-represented pathways. We used normalized count reads, i.e. counts per million and log2 transformed, and performed a pathway enrichment analysis with Ingenuity pathway analysis (IPA) software.

### Statistics

Statistical analysis was performed by using GraphPad Prism 6. Statistical differences were examined by applying one and two-way analysis of variance tests (ANOVAs) followed by Tukey’s Multiple Comparison Test. A *p* value of less than 0.05 (P < 0.05) was considered statistically significant. Data are presented as the mean ± standard deviation (SD) or ± standard error of the mean (SEM) of 5–7 retinas per group.

### Data availability

All data generated or analysed during this study are included in this published article (and its Supplementary Information files).

## Electronic supplementary material

Supplementary Information
