# Supplementary material for: Resistance to retinopathy development in obese, diabetic and hypertensive ZSF1 rats: an exciting model to identify protective genes
Source: Sci Rep. 2018 Aug 9;8:11922. doi: 10.1038/s41598-018-29812-w (PMC6085379; doi:10.1038/s41598-018-29812-w)
Supplement: Supplementary file 1 — Supplementary Information [file 41598_2018_29812_MOESM1_ESM.pdf]

Resistance to retinopathy development in obese, diabetic and hypertensive ZSF1 rats: an exciting model to identify protective genes.

Vincenza Caolo*[1], Quentin Roblain[2,3], Julie Lecomte[3], Paolo Carai[1], Linsey Peters[2], Ilona Cuijpers[1,2], Emma Louise Robinson[2], Kasper Derks[4], Jurgen Sergeys[5], Noël Agnès[3], Elizabeth A.V. Jones[1], Lieve Moons[5], Stephane Heymans[1,2, 6]

[1] Department of Cardiovascular Sciences, Centre for Molecular and Vascular Biology, KU Leuven, Belgium.
[2] Department of Cardiology, CARIM School for Cardiovascular Diseases Faculty of Health, Medicine and Life Sciences, Maastricht University, The Netherlands.
[3] Laboratory of Tumor and Development Biology, GIGA-Cancer, University of Liège, Liège, Belgium.
[4] Department of Genetics and Cell Biology, CARIM School for Cardiovascular Diseases Faculty of Health, Medicine and Life Sciences, Maastricht University, The Netherlands.
[5] Laboratory of Neural Circuit Development and Regeneration, Animal Physiology and Neurobiology Section, Department of Biology, KU Leuven, Leuven, Belgium.
[6] The Netherlands Heart Institute, Nl-HI, Utrecht, The Netherlands

Corresponding Author:
Vincenza Caolo; Centre for Molecular and Vascular Biology, KU Leuven, Belgium.
Email: Vincenza.caolo@kuleuven.be. Tel. +32 16 37 71 25

Supplementary Figure and Table Legends

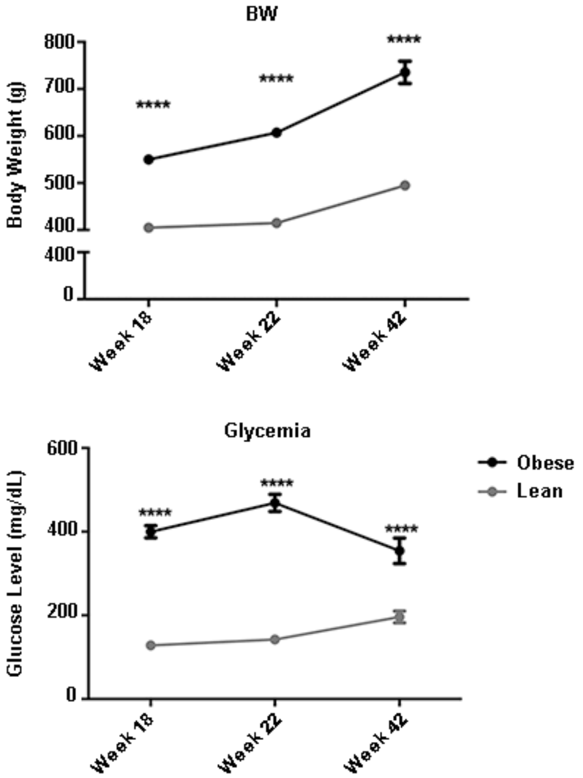

Supplementary Figure 1 Body weight and glucose level measured in lean and obese ZSF1 rats at 18, 22 and 42 weeks. All values are mean ± SEM, ****P<0.05.

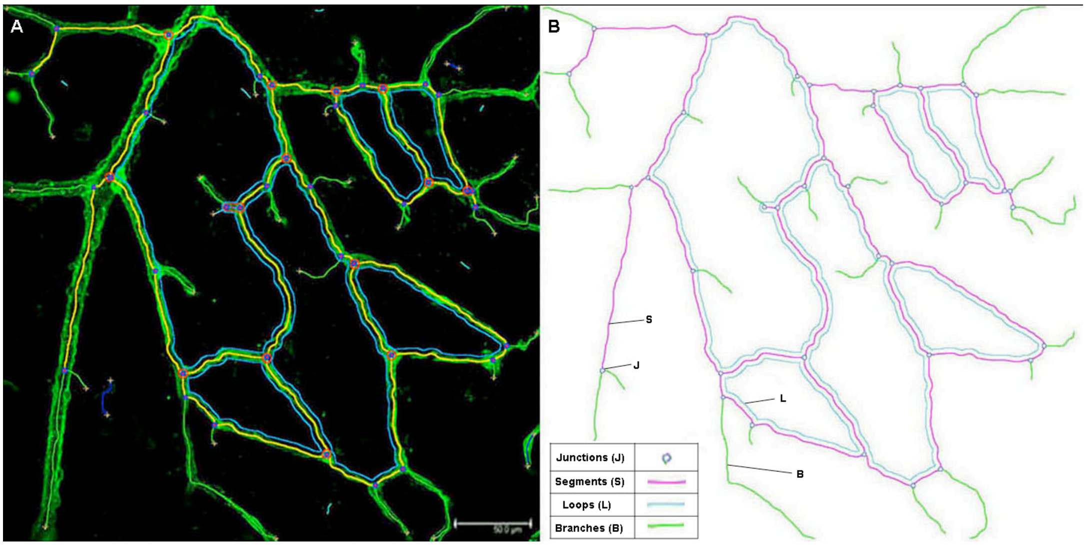

Supplementary Figure 2 Example of vascular network analysed by Angiogenesis Analyzer (**A**) and map (**B**) including junctions, segments, loops and branches. Scale = 50µm. Junctions (J) are indicated as dark blue circles and are the meeting point of several segments. Segments (S) are indicated in fuchsia and are vessels between two junctions. Loops (L) are indicated in light blue and are structures enclosed by segments. Branches (B) are vessels on segments that are not connected on one end.

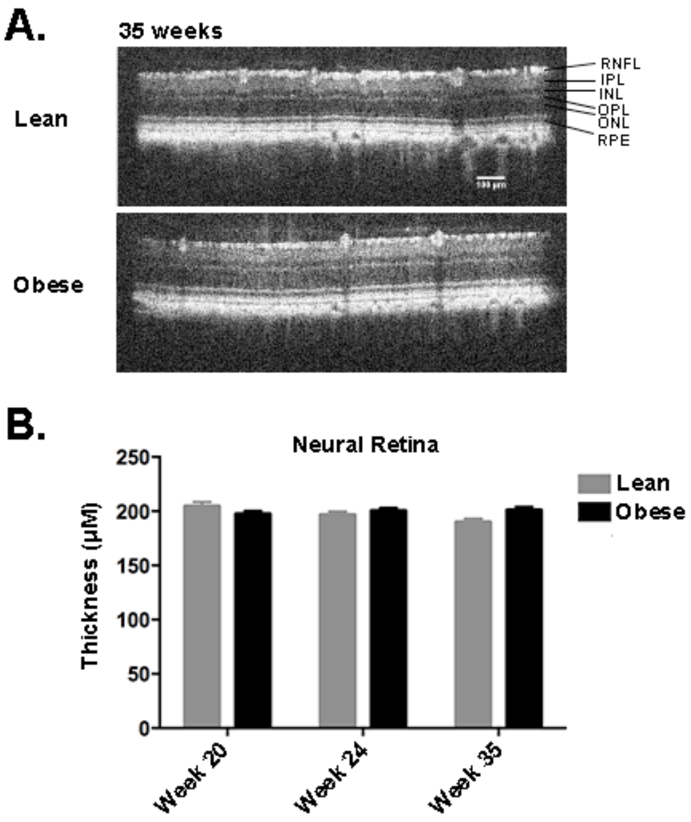

Supplementary Figure 3 Spectral domain optical coherence tomography (SD-OCT) analysis performed in lean and obese ZSF1 rats. (**A**) Representative SD-OCT pictures show no difference in morphology of the neural retina in Lean and Obese rats 35 weeks old. RNFL, retinal nerve fiber layer; IPL, inner plexiform layer; INL, inner nuclear layer; OPL, outer plexiform layer; ONL, outer nuclear layer; RPE, retinal pigment epithelium. Scale bar: 100 μm; (**B**) Analysis of SD-OCT images revealed a similar thickness of the neural retina in Lean and Obese rats at 20, 24 and 35 week, respectively.

Table 1 Lean vs Obese 6 week old

Top canonical pathways		
**Name**	**p-value**	**Overlap**
Allograft Rejection Signaling	3.64E-08	24.5% 12/49
OX40 Signaling Pathway	5.90E-08	21.3% 13/61
Cdc42 Signaling	8.14E-07	13.3% 17/128
Antigen Presentation Pathway	7.06E-05	26.1% 6/23
EIF2 Signaling	9.80E-05	9.0% 18/201

Supplementary Table 1 Top canonical pathways significantly enriched by Ingenuity Pathway Analysis (IPA) from the differentially expressed genes found between lean vs obese 6 weeks old.

Table 2 Lean vs Obese 42 week old

Top canonical pathways		
**Name**	**p-value**	**Overlap**
Antigen Presentation Pathway	8.67E-09	56,5% 13/23
OX40 Signaling Pathway	2.23E-08	34.4% 21/61
Allograft Rejection Signaling	7.14E-08	36.7% 18/49
Complement System	1.77E-05	35,3% 12/34
Cdc42 Signaling	4.12E-05	20.03% 26/128

Supplementary Table 2 IPA revealed the top 5 canonical pathways differentially expressed in retina isolated from lean vs obese 42 weeks old.

Table 3 Lean 6 vs Lean 42 week old

Top canonical pathways		
**Name**	**p-value**	**Overlap**
EIF2 Signaling	1.24E-06	6,5% 13/201
Calcium Transport I	2.05E-04	30% 3/10
B Cell Development	2.28E-04	16% 4/25
Hepatic Fibrosis / Hepatic Stellate Cell Activation	1.41E-03	4.6% 8/174
nNOS Signaling in Skeletal Muscle Cells	1.07E-02	15.4% 2/13

Supplementary Table 3 IPA revealed the top 5 canonical pathways differentially expressed in retina isolated from lean 6 vs lean 42 weeks old.

Table 4 Obese 6 vs Obese 42 week old

Top canonical pathways		
**Name**	**p-value**	**Overlap**
Leptin Signaling in Obesity	4.44E-03	3.6% 3/84
Coagulation System	7.91E-03	5.9% 2/34
cAMP-mediated signaling	1.06E-02	1.8% 4/214
Thyronamine and Iodothyronamine Metabolism	1.18E-02	33.3% 1/3
Thyroid Hormone Metabolism I (via Deiodination)	1.18E-02	33.3% 1/3

Supplementary Table 4 IPA revealed the top 5 canonical pathways differentially expressed in retina isolated from obese 6 vs obese 42 weeks old.
